# *Lyz1*-Expressing Alveolar Type II Cells Contribute to Lung Regeneration

**DOI:** 10.70322/jrbtm.2025.10011

**Published:** 2025-11-27

**Authors:** Yinshan Fang, Kangchen Li, Bryan Ding, Nan Gao, Jie Sun, Jianwen Que

**Affiliations:** 1 Columbia Center for Human Development and Division of Digestive and Liver Disease, Department of Medicine, Vagelos College of Physicians and Surgeons, Columbia University Irving Medical Center, New York, NY 10032, USA; 2 Collaborative Innovation Center for Chinese Medicine and Respiratory Diseases Co-Constructed by Henan Province & Education Ministry of P.R. China, Henan Key Laboratory of Chinese Medicine for Respiratory Disease, Academy of Chinese Medical Sciences, Henan University of Chinese Medicine, Zhengzhou 450003, China; 3 Department of Pharmacology, Physiology, and Neurosciences, Rutgers Health-New Jersey Medical School, Newark, NJ 07103, USA; 4 Beirne B. Carter Center for Immunology Research and Division of Infectious Disease and International Health, Department of Medicine, University of Virginia, Charlottesville, VA 22908, USA

**Keywords:** *Lyz1*, AT2 subpopulation, Lung regeneration, scRNA-seq

## Abstract

The alveolar units, composed of alveolar epithelial type II cells (AT2) and type I cells (AT1), are essential for efficient gas exchange. While AT2 cells are known to play critical roles in alveolar homeostasis and regeneration, the contribution of heterogeneous AT2 cells to lung repair remains poorly understood. Here, we identified a distinct AT2 subpopulation that exclusively expressed Lysozyme 1 (*Lyz1*) through single-cell RNA sequencing (scRNA-seq) analyses. Cell fate mapping revealed that the *Lyz1*^*CreERT2*^ mouse strain specifically labeled *Lyz1*-expressing AT2 cells *in vivo* at homeostasis. Following lung injury, *Lyz1*^+^ AT2 cells expanded and contributed to alveolar regeneration by generating both self-renewing AT2 cells and differentiating AT1 cells. We further observed the emergence of *de novo Lyz1*-expressing cells in the airways after lung injury. Additionally, *Lyz1*^+^ AT2 cells displayed significantly enhanced proliferative capacity compared with general bulk AT2 cells in 3D organoid cultures. These findings define *Lyz1*^+^ AT2 cells as a previously unrecognized progenitor population, expanding the paradigm of alveolar regeneration and providing insight into how epithelial diversity supports lung regeneration.

## 1. Introduction

The lung is a highly specialized organ responsible for efficient gas exchange [1,2]. It’s essential to restore alveolar architecture and function through regeneration to preserve respiratory capacity after lung injury [3,4]. Multiple epithelial populations have been reported to contribute to alveolar repair. Following alveolar damage, airway club cells can migrate into the alveolar region and differentiate into AT2 cells [5–8]. Bronchioalveolar stem cells (BASCs), positioned at the bronchioalveolar duct junctions, co-expressing the club cell marker *Scgb1a1* and the AT2 cell marker surfactant protein C (*Sftpc* or *SPC*), have been shown to give rise to AT2 cells following injury [7,9]. Within the alveolus, AT2 cells serve as stem cells, capable of self-renewing to generate new AT2 cells and differentiating into AT1 cells, thereby restoring the epithelial barrier [7,10]. Although it remains controversial, AT1 cells have been reported to dedifferentiate into AT2 cells in certain contexts, such as pneumonectomy or hyperoxia-induced lung injury [7,11,12].

Mounting evidence has indicated that AT2 cells display substantial heterogeneity with distinct subsets contributing to lung regeneration [13–17]. Recent studies showed that Axin2^+^ AT2 cells located in close proximity to Wnt-secreting fibroblasts serve as progenitors during both homeostasis and regeneration [13,14]. Further studies indicated that this Axin2^+^ AT2 cell subpopulation expresses the cell membrane protein TM4SF1 [14]. In addition, the cell surface glycoprotein CD44 is also highly expressed in a subset of AT2 cells, which seem more proliferative and have the potential to generate more versatile cells than CD44^low^ AT2 cells [18]. More recently, long-term lineage tracing of AT2 cells revealed two independent subsets, faster-cycling and slower-cycling cells in the mouse lungs [17]. The faster-cycling AT2 cell pool seems derived from the Il1r1^+^ AT2 subpopulation that generates damage-associated transient progenitors (DATP) upon IL-1β signaling activation during alveolar regeneration [15,17]. Despite these insights, the relationships among distinct AT2 subpopulations remain unclear, and additional AT2 subsets may exist. Furthermore, many of the subsets described above have been defined primarily through functional assays, with limited resolution at the single-cell transcriptomic level.

scRNA-seq has transformed our ability to dissect epithelial diversity, uncovering previously unrecognized cell states and lineages [19–22]. However, application of this approach to fully chart AT2 heterogeneity has been limited, and the spectrum of AT2 progenitor identities in regeneration remains incomplete. Here, we leveraged scRNA-seq to uncover an AT2 subpopulation defined by exclusive *Lyz1* expression. A genetic cell fate-mapping mouse model demonstrated that these *Lyz1*^+^ AT2 cells contributed to lung regeneration after injury. Additionally, a 3D *in vitro* organoid assay revealed that *Lyz1*^+^ AT2 cells exhibited a higher proliferative capacity than bulk AT2 cells. These findings established *Lyz1*^+^ AT2 cells as a novel progenitor population that expands the cellular paradigm of alveolar regeneration and provide a foundation for understanding how epithelial diversity drives lung repair.

## 2. Results

### 2.1. Lyz1 Is Expressed in a Subpopulation of AT2 Cells

**Figure 1.**
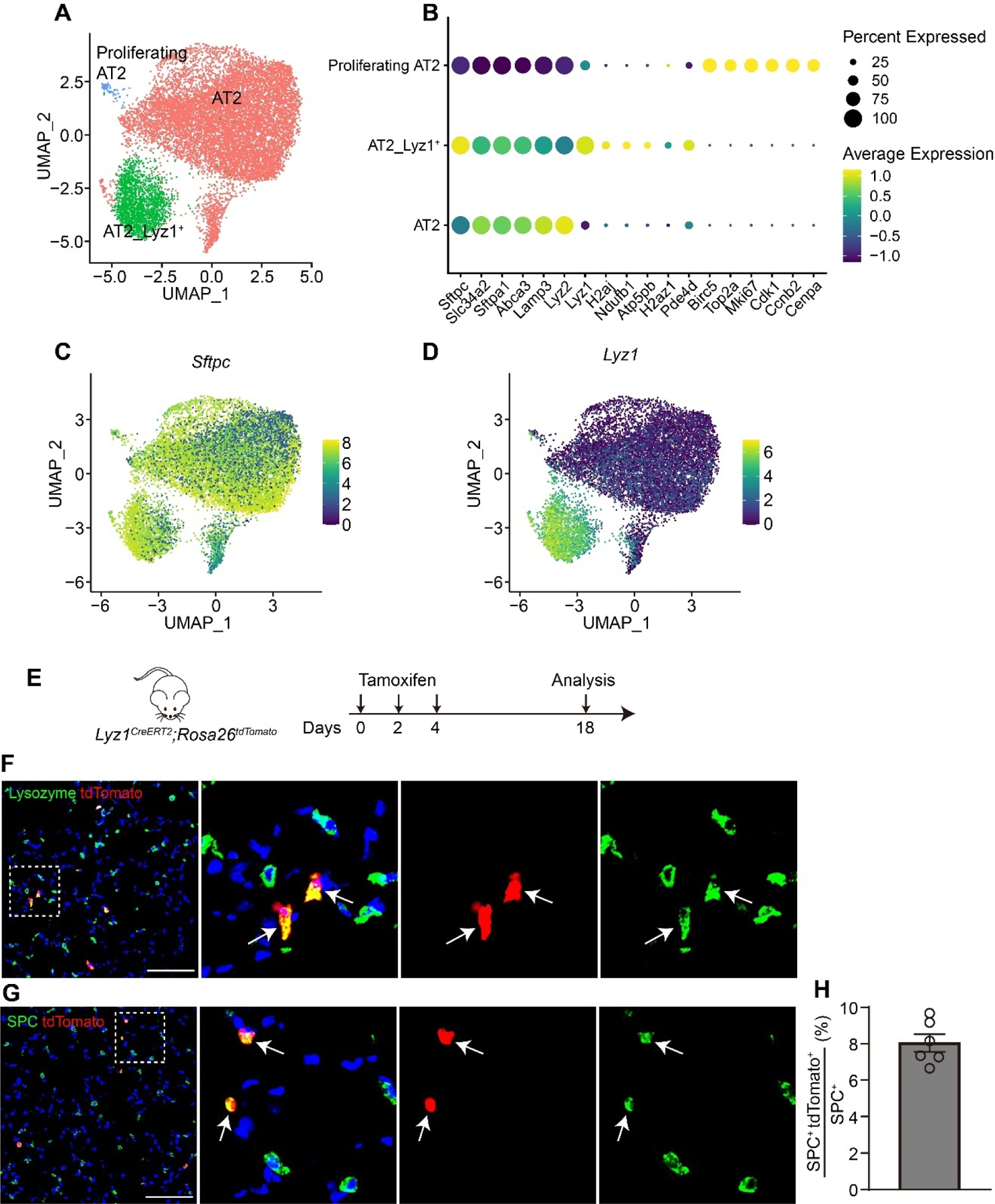
*Lyz1* marks an AT2 cell subpopulation. (**A**) UMAP plot showing AT2 cell populations in adult mouse lungs. Re-analysis of the publicly available datasets GSE171571, GSE132910, GSE138585 and GSE202226. (**B**) Dot plot showing the representative markers for each AT2 cell population. (**C**,**D**) UMAP plot showing the expression of *Sftpc* (**C**) and *Lyz1* (**D**) in adult mouse AT2 cell populations. (**E**) Schematic of tamoxifen injection of *Lyz1*^*CreERT2*^;*R26*^*tdT*^ mice. (**F**) Representative images showing Lysozyme^+^tdTomato^+^ (arrows) cells in lung tissues. Magnified image of dashed square frame showing on the right. (**G**) Representative images showing SPC^+^tdTomato^+^ (arrows) cells in lung tissues. Magnified image of dashed square frame showing on the right. (**H**) Quantification analysis showing the percentage of SPC^+^tdTomato^+^ cells in total SPC^+^ AT2 cells (*n* = 6). Data are representative of at least three independent experiments. Data are mean ± SEM. Scale bars: 100 μm.

To better characterize the heterogeneity of AT2 cells, we re-analyzed mouse lung epithelial cells from publicly available scRNA-seq datasets [22–25] (Figure S1A,B). Re-clustering of AT2 cell population revealed three distinct subpopulations at homeostasis (Figures 1A–D and S1C–E). Notably, one subpopulation exclusively expressed the lysozyme C-1 encoding gene *Lyz1* (Figure 1B,D, and Supplemental Table S1), which also marked intestinal Paneth cells [26], whereas lysozyme C-2 encoding gene *Lyz2* was broadly expressed across nearly all AT2 cells (Figure 1B). Further pathway analyses revealed that rRNA processing, ribosome assembly and translation pathways were enriched in *Lyz1*^+^ AT2 subpopulation (Figure S1F,G). To investigate the lineage potential of *Lyz1*-expressing cells *in vivo*, we generated *Lyz1*^*CreERT2*^; *R26*^*tdTomato*^ (*R26*^*tdT*^) mice. Following three intraperitoneal injections of tamoxifen (Tmx), a small fraction of tdTomato-positive (tdT^+^) cells were detected in the adult lungs (Figure 1E,F). Immunostaining confirmed that all tdT^+^ cells co-expressed Lysozyme (Figure 1F) and SPC (Figure 1G), verifying their identity as AT2 cells. Quantitative analysis showed that approximately 8% AT2 cells were tdT^+^ (Figure 1H), demonstrating that *Lyz1*-expressing cells represent a distinct subpopulation within the AT2 cell lineage.

### 2.2. Lyz1^+^ AT2 Cells Expand and Differentiate into AT1 Cells in the Regenerated Lungs

**Figure 2.**
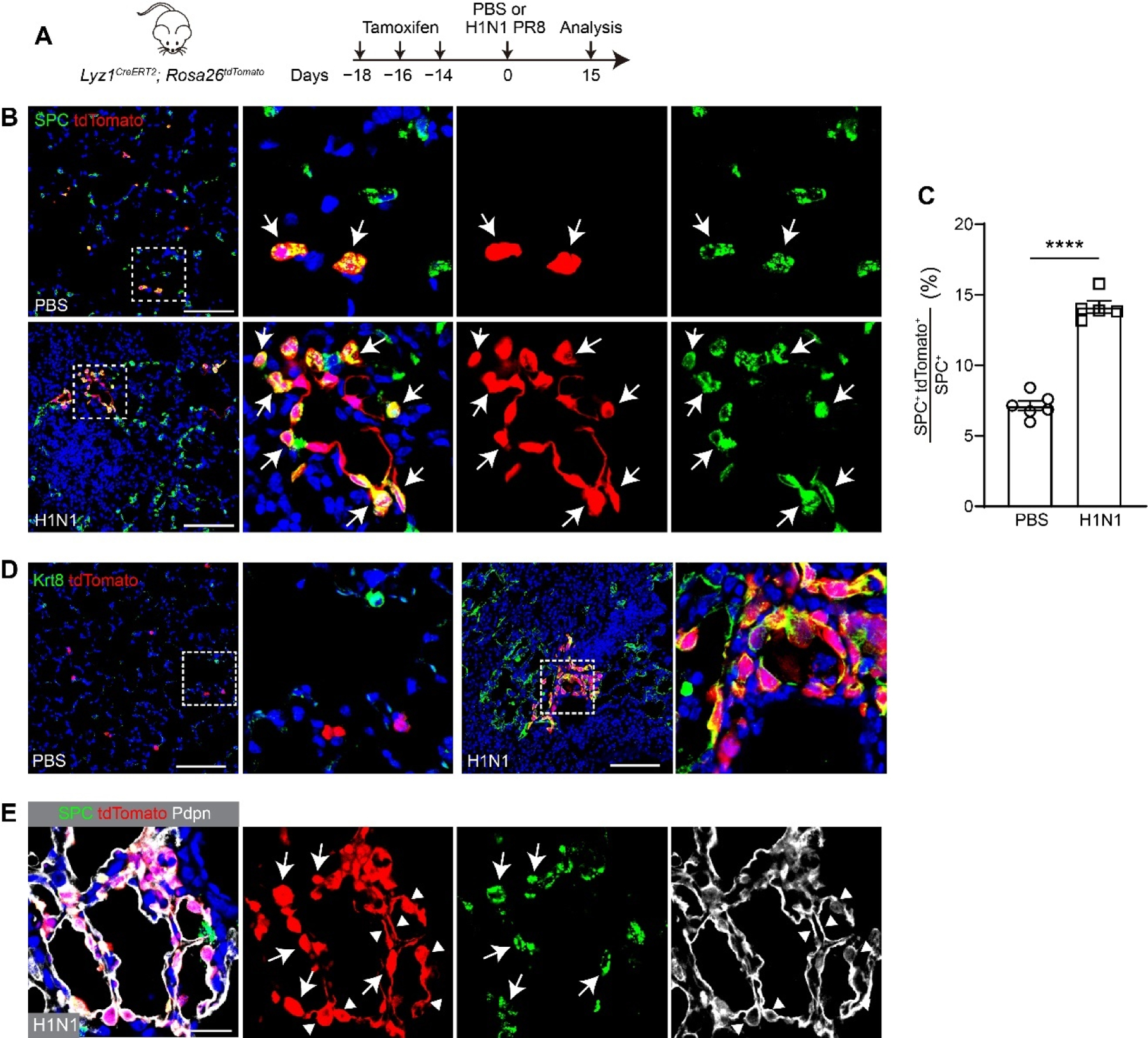
*Lyz1*^*CreERT2*^ lineage-labeled AT2 cells contribute to lung regeneration following H1N1 influenza challenge. (**A**) Schematic of tamoxifen injection and H1N1 PR8 influenza infection of *Lyz1*^*CreERT2*^;*R26*^*tdT*^ mice. (**B**) Representative images showing SPC^+^tdTomato^+^ cells (arrows) in lung tissues. Magnified image of dashed square frame showing on the right. (**C**) Quantification analysis showing the percentage of SPC^+^tdTomato^+^ cells in total SPC^+^ AT2 cells (*n* = 6). (**D**) Representative images showing Krt8^+^tdTomato^+^ cells in lung tissues. Magnified image of dashed square frame showing on the right. (**E**) Representative images showing SPC^+^tdTomato^+^ cells (arrows) and Pdpn^+^tdTomato^+^ cells (triangles) in lung tissues. Data are mean ± SEM. ****: *p* < 0.0001. Scale bars: 100 μm.

To determine whether *Lyz1*^+^ AT2 cells contribute to lung regeneration following injury, *Lyz1*^*CreERT2*^;*R26*^*tdT*^ mice were administered three doses of Tmx and subsequently challenged with H1N1 PR8 influenza virus (Figure 2A). Notably, clusters of tdT^+^ cells were observed in lungs at 15 days post-infection (dpi), with the majority of tdT^+^ cells expressing SPC (Figure 2B). However, only sporadic individual tdT^+^ AT2 cells were detected in control mice that were treated with PBS (Figure 2B). Consistently, we observed a significant increase in the proportion of tdT^+^ AT2 cells in virus-injured lungs compared to PBS-treated controls (14.13 ± 0.44% *vs*. 7.13 ± 0.34%, *p* < 0.0001) (Figure 2C). Furthermore, *Lyz1*^*CreERT2*^;*R26*^*tdT*^ mice were subjected to bleomycin challenge following Tmx administration (Figure S2A). An increased number of tdT^+^ cells was detected in bleomycin-injured lungs compared to saline-treated controls. Similar to the virus-infected lungs, the tdT^+^ cells co-expressing SPC formed clusters (Figure S2B). Together, these results suggest that *Lyz1*^+^ AT2 cells actively expand and contribute to the AT2 cell pool during injury and repair.

Previous studies have demonstrated that AT2 cells function as progenitors, giving rise to AT1 cells during lung regeneration [10,27–29]. This transition involves an intermediate cell state characterized by the expression of markers such as Krt8 and Cldn4 [15,30,31]. To determine whether *Lyz1*^+^ AT2 cells follow this differentiation pathway, we examined the expression of Krt8 in the injured lungs. Krt8^+^ cells were present in the peripheral lungs of H1N1 virus- or bleomycin-challenged mice but were rarely seen in control lungs (Figures 2D and S2C). Notably, a subset of Krt8^+^ cells co-expressed tdT and were only found in the injured but not the control lungs, indicating that *Lyz1*^+^ AT2 cells gave rise to Krt8^+^ transitional cells during regeneration. tdT^+^ cells in the injured lungs also included elongated AT1 cells (Pdpn^+^) (Figures 2E and S2D). Taken together, our data demonstrate that *Lyz1*^+^ AT2 cells not only expand but also differentiated into AT1 cells via a transitional Krt8^+^ intermediate cell state, thereby contributing to alveolar regeneration following injury.

### 2.3. De Novo Lyz1-Expressing Cells Emerged Following Lung Injury

**Figure 3.**
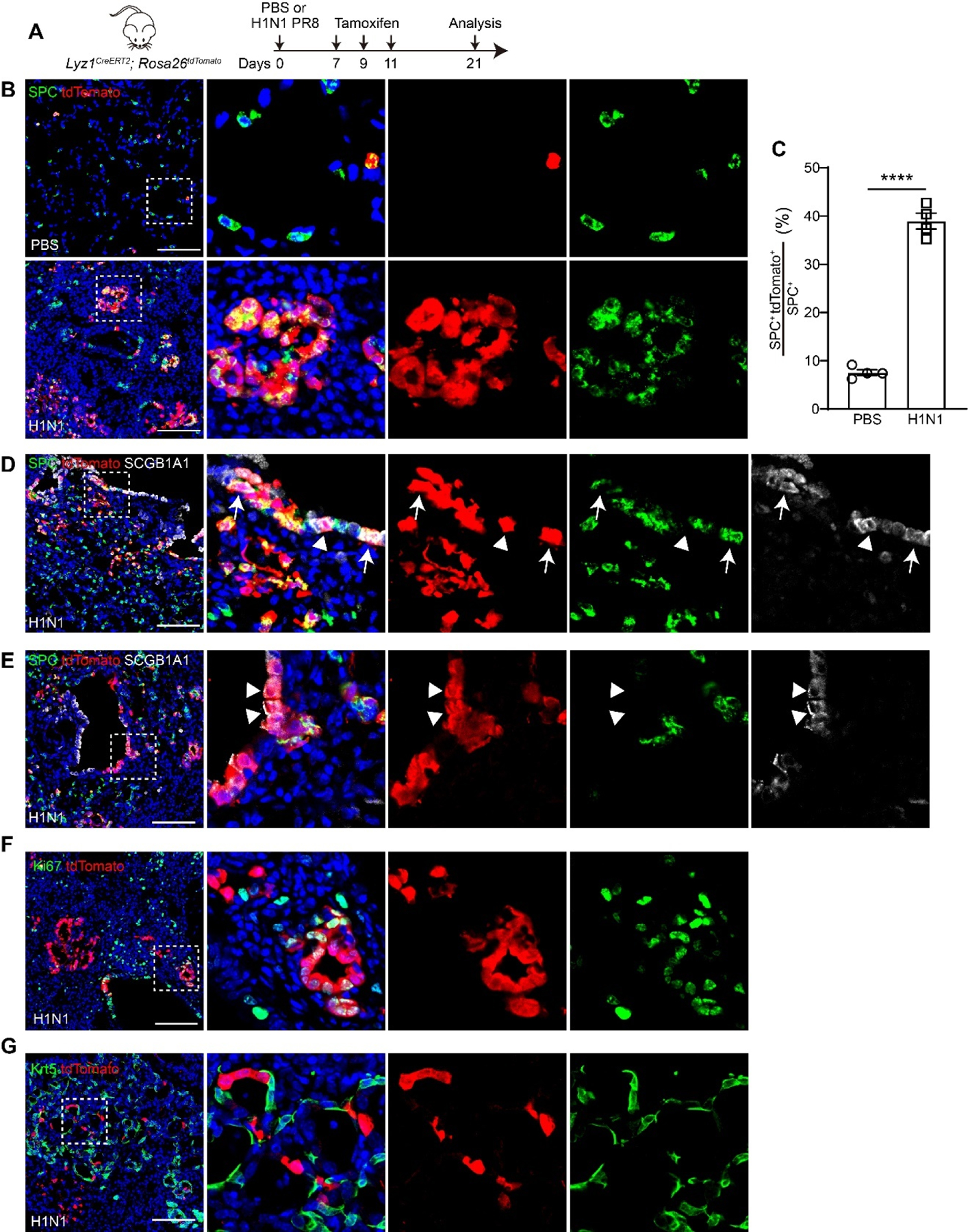
*De novo Lyz1*-expressing cells emerge following H1N1 influenza challenge. (**A**) Schematic of H1N1 PR8 influenza infection and tamoxifen injection of *Lyz1*^*CreERT2*^;*R26*^*tdT*^ mice. (**B**) Representative images showing SPC^+^tdTomato^+^ cells in lung tissues. (**C**) Quantification analysis showing the percentage of SPC^+^tdTomato^+^ cells in total SPC^+^ AT2 cells (*n* = 4). (**D**,**E**) Representative images showing expression of SCGB1A1, SPC and tdTomato in lung tissues. Arrows indicate SPC^+^SCGB1A1^+^tdTomato^+^ cells. Triangles indicate SCGB1A1^+^tdTomato^+^ cells. (**F**) Representative images showing Ki67^+^tdTomato^+^ cells in lung tissues. (**G**) Representative images showing immunostaining of Krt5 and tdTomato. (**B**,**D**–**G)**: Magnified image of dashed square frame showing on the right. Data are representative of at least three independent experiments. Data are mean ± SEM. ****: *p* < 0.0001. Scale bars: 100 μm.

To investigate whether *Lyz1* is expressed in the newly regenerated cells following injury, we challenged *Lyz1*^*CreERT2*^;*R26*^*tdT*^ mice with H1N1 virus, followed by administration of three doses of Tmx (Figure 3A). Remarkably, ring-like clusters of AT2 cells co-expressing tdT were observed in H1N1 virus-infected lungs (Figure 3B). The numbers of tdT^+^SPC^+^ AT2 cells increased in virus-injured lungs compared to PBS-treated controls (38.94 ± 1.64% *vs*. 7.55 ± 0.60%, *p* < 0.0001, Figure 3C). Moreover, tdT^+^ cells were also present in the intrapulmonary airways of virus-infected but not control mice (Figure 3D,E). Notably, these tdT^+^ cells in the airways expressed SPC (Figure 3D), consistent with a previous report that severe lung injury induces ectopic expression of the AT2 marker in airway epithelial cells [32]. Some tdT^+^ cells in the airways expressed the club cell marker SCGB1A1 or both SPC and SCGB1A1 (Figure 3D,E). Furthermore, tdT^+^ cells exhibited highly proliferative activity, as indicated by co-expression of the proliferation marker Ki67 (Figure 3F). Viral infection causes severe lung injury and induces aberrant basal-like cells in distal lungs [33–35]. However, aberrant Krt5^+^ basal-like cells rarely co-expressed tdT in virus-challenged lungs (Figure 3G). Collectively, these findings demonstrate that *Lyz1* expression is induced in newly regenerated AT2 cells and club cells, but not aberrant Krt5^+^ basal-like cells, following lung injury, and that these cells retain the capacity to proliferate during regeneration.

### 2.4. Lyz1^+^ AT2 Cells Self-Renew and Differentiate in Cultured Organoids

**Figure 4.**
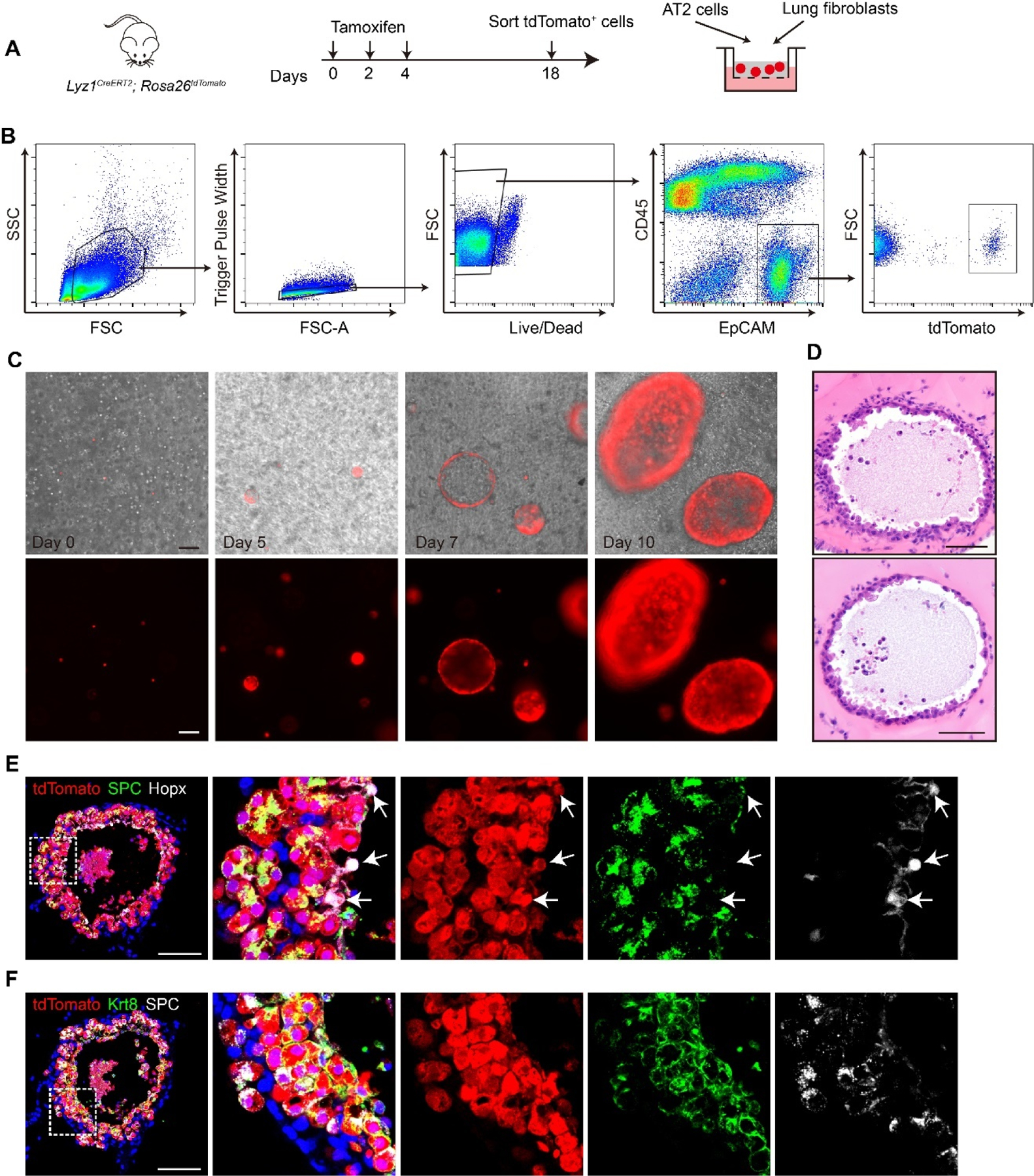
*Lyz1*^+^ AT2 cells form organoids in 3D culture system. (**A**) Schematic of tamoxifen injection of *Lyz1*^*CreERT2*^;*R26*^*tdT*^ mice, isolation of tdTomato^+^ AT2 cells and co-culture with lung fibroblasts. (**B**) Gating strategy to sort tdTomato^+^ AT2 cells from the lungs of *Lyz1*^*CreERT2*^;*R26*^*tdT*^ mice. (**C**) Representative images of organoids at the indicated time points. (**D**) Representative images of H&E staining for organoids. (**E**) Representative images showing immunostaining of SPC, Hopx and tdTomato in organoids. Arrows indicate Hopx^+^tdTomato^+^ cells. (**F**) Representative images of immunostaining for SPC, Krt8, and tdTomato in organoids. Magnified image of dashed square frame showing on the right. Data are representative of at least three independent experiments. Scale bars: 100 μm.

We next tested whether *Lyz1*^+^ AT2 cells are able to form 3D organoids. tdT^+^ AT2 cells were isolated by Fluorescence-Activated Cell Sorting (FACS) from the lungs of adult *Lyz1*^*CreERT2*^;*R26*^*tdT*^ mice treated with three doses of Tmx. The sorted cells were co-cultured with adult mouse lung fibroblasts in a 3D Matrigel-based organoid system (Figure 4A,B). Single tdT^+^ cells proliferated to form cell clusters and developed into spherical organoids (Figure 4C), indicating the robust self-renewal capacity of *Lyz1*^+^ AT2 cells. Histological analysis revealed that the organoids maintained normal cellular morphology and nuclear structure (Figure 4D). tdT^+^ cells within the organoids were detected, while a few tdT^−^ cells exhibiting elongated morphology, presumably lung fibroblasts, were localized to the outer layer of the organoid (Figure 4D,E). In addition, immunostaining showed that the tdT^+^ cells in organoids expressed SPC, confirming their AT2 cell identities (Figure 4E). Notably, a subset of tdT^+^ cells located in the organoid inner region expressed Hopx, suggesting the differentiation of *Lyz1*^+^ AT2 cells into AT1 cells (Figure 4E). We also detected numerous tdT^+^ cells that expressed KRT8 but not SPC (Figure 4F), supporting the presence of transitional states during AT2−AT1 differentiation. Therefore, our findings indicate that *Lyz1*^+^ AT2 cells are capable of forming organoids *in vitro* with self-renewal and differentiation potential.

### 2.5. Lyz1^+^ AT2 Cells Exhibit Enhanced Proliferative Capacity

**Figure 5.**
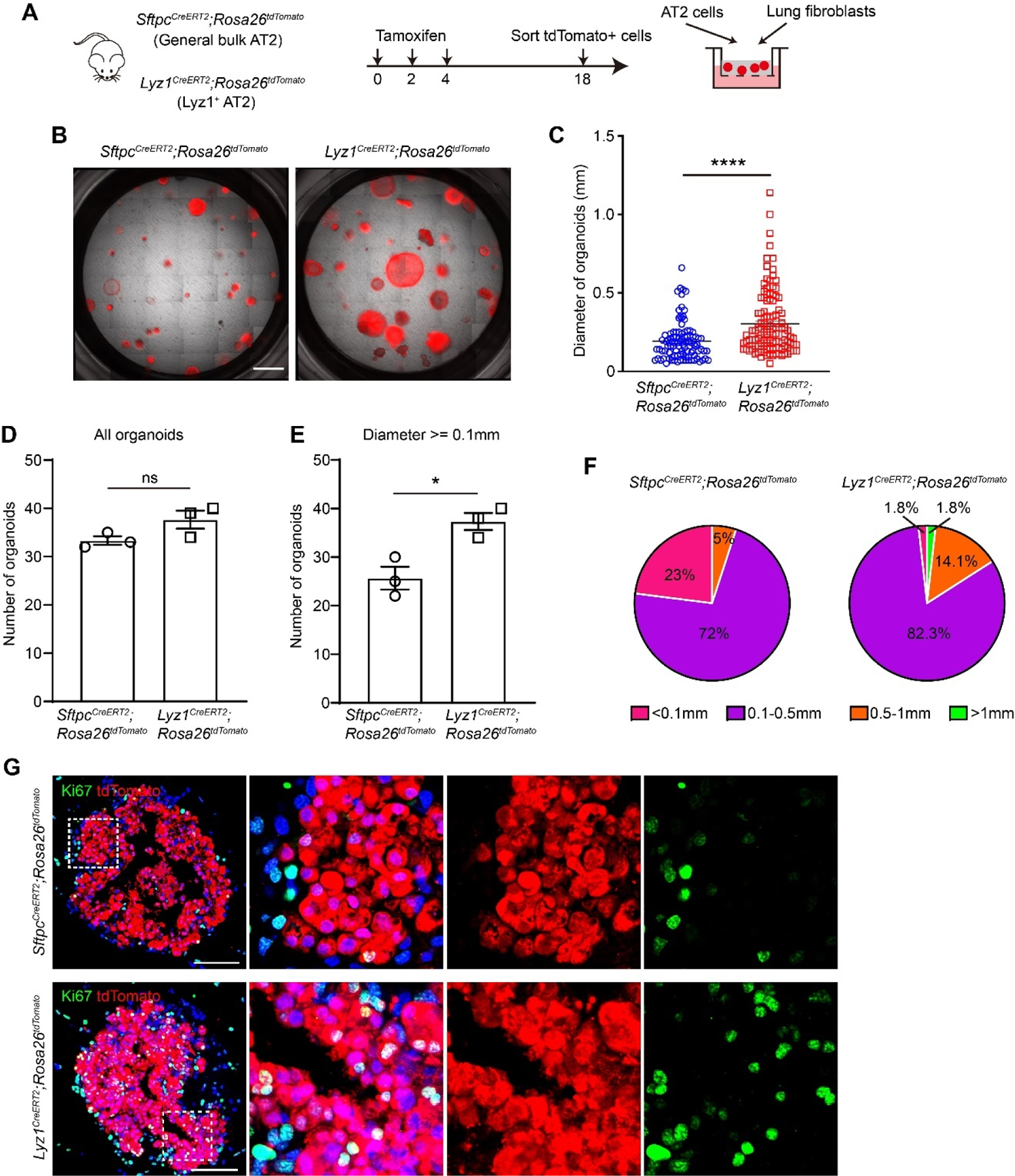
*Lyz1*^+^ AT2 cells exhibit high proliferative capacity. (**A**) Schematic of tamoxifen injection of *Lyz1*^*CreERT2*^;*R26*^*tdT*^ and *Sftpc*^*CreERT2*^;*R26*^*tdT*^ mice, isolation of tdTomato^+^ general AT2 and *Lyz1*^+^ AT2 cells, and co-culture with lung fibroblasts. (**B**) Representative images of organoids derived from AT2 cells of *Lyz1*^*CreERT2*^;*R26*^*tdT*^ and *Sftpc*^*CreERT2*^;*R26*^*tdT*^ mice. (**C**) Quantification of the diameter of each organoid. (**D**,**E**) Quantification of the number of total organoids (**D**) and organoids whose diameter is larger than 0.1 mm. (**E**) *n* = 3. (**F**) Pie chart showing the percentages of different organoid sizes as indicated by colors. (**G**) Representative images showing immunostaining of Ki67 and tdTomato in organoids established with cells isolated from the lungs of *Lyz1*^*CreERT2*^;*R26*^*tdT*^ and *Sftpc*^*CreERT2*^;*R26*^*tdT*^ mice. Magnified image of dashed square frame showing on the right. Data are representative of at least three independent experiments. Data are mean ± SEM. ns: no significance. *: *p* < 0.05. ****: *p* < 0.0001. Scale bars: 100 μm.

To investigate whether *Lyz1*^+^ AT2 cells have increased self-renewal potential compared to general bulk AT2 cells, we cultured these two cell populations in a 3D organoid culture system. FACS isolated general bulk AT2 cells and *Lyz1*^+^ AT2 cells from *Sftpc*^*CreERT2*^;*R26*^*tdT*^ and *Lyz1*^*CreERT2*^;*R26*^*tdT*^ mice, respectively, and co-cultured with mouse lung fibroblasts in Matrigel (Figure 5A). Both populations were able to establish tdT^+^ organoids (Figure 5B). Notably, *Lyz1*^+^ AT2 cells generated significantly larger organoids compared to general bulk AT2 cells (Figure 5B,C). Although the total number of organoids was comparable between these two groups, the frequency of large organoids (diameter > 0.5 mm) was markedly enriched in the *Lyz1*+ AT2 group (Figure 5D,E). While approximately 23% organoids derived from general AT2 cells were small (diameter < 0.1 mm), 1.8% organoids in the *Lyz1*^+^ AT2 group fell into this category (Figure 5F). Consistently, large organoids (>0.5 mm) were predominantly derived from *Lyz1*^+^ AT2 cells (Figure 5F). Consistent with the observed differences in organoid size, we detected a significantly higher numbers of tdT^+^Ki67^+^ proliferating cells within *Lyz1*^+^ AT2 cells-derived organoids (Figure 5G). Together, these results establish that *Lyz1*^+^ AT2 cells possess a marked intrinsic growth advantage and form large organoids *in vitro*, highlighting their robust self-renewal potential.

## 3. Discussion

The heterogeneity of AT2 cells under homeostatic conditions remains incompletely understood. By reanalyzing publicly available scRNA-seq datasets [22,23], we discovered that the lysozyme C–1 encoding gene *Lyz1* is exclusively expressed in a distinct AT2 cell subpopulation in normal mouse lungs. *Lyz1* was also reported to encode lysozyme in Intestinal Paneth cells to process bacterial cell walls [36]. Previous study indicated that ablation of *Lyz1* protected mice from experimental colitis [37]. Notably, AT2 cells also express *Lyz2*, which encodes lysozyme C–2. It remains unknown whether *Lyz2* compensates for the loss of *Lyz1* in a loss-of-function context. Along these lines, it would be interesting to determine whether lysozyme C-1, secreted by *Lyz1*^+^ AT2 cells, plays a role in inflammatory lung diseases. Cell fate-mapping using *Lyz1*^*CreERT2*^ mouse strain further revealed that approximately 10% AT2 cells were labeled. Recently, several studies employing cell fate-mapping and flow cytometry have highlighted the heterogeneity of AT2 cells at homeostasis [13,14,16,18]. For instance, Wnt-responsive Axin2^+^ AT2 cells comprised 1~20% of the AT2 population in two independent studies [13,14]. In addition, about 3% AT2 cells expressed a high level of CD44 [18], which has been shown to mark stem cells in multiple tissues [38,39]. CD44^high^ AT2 cells are located at the perivascular regions in the mouse lungs [25]. In contrast, we did not observe a specific anatomical location for *Lyz1*^+^ AT2 cells. Moreover, an AT2 subpopulation characterized by high PD-L1/CD274 and low SPC expression has also been shown to act as progenitor cells [16]. While the *Lyz1*^+^ AT2 subpopulation stands out as a distinct entity in our scRNA-seq analysis, we did not detect subpopulations defined exclusively by Axin2, CD44 or CD274 expression. Therefore, further work is needed to determine the relationship among these different AT2 subpopulations.

AT2 cells are well established as stem cells that contribute to lung regeneration [7,10]. Here, we demonstrated that *Lyz1*^+^ AT2 cells are able to expand and differentiate into AT1 cells following both influenza- and bleomycin-induced lung injury. Additionally, we observed *de novo* emergence of *Lyz1*-expressing AT2 cells after injury, consistent with previous findings that quiescent AT2 cells acquire stem/progenitor-like properties in response to damage [13,40]. It has been shown that while only a small fraction of AT2 cells express Sca-1 in normal lung tissues, 30–70% AT2 cells express Sca-1 following *Pseudomonas aeruginosa* infection [40]. Similarly, Axin2^+^ AT2 cells have been reported to represent 1% the AT2 pool at homeostasis but expand to about 73% after diphtheria toxin-induced lung injury, largely derived from pre-existing Axin2^−^ AT2 cells [13]. More recently, Liu et al. combined novel intersectional genetics with scRNA-seq to show that BASC cells generate heterogeneous AT2 subpopulations, including Chil1^high^, Cxcl15^high^ or Ereg^high^ AT2 subpopulations following bleomycin challenge [7]. Whether these subpopulations originate from club cells, pre-existing AT2 cells, or other intermediates remains unresolved. Along this line, the source of those newly generated *Lyz1*^+^ AT2 cells remains to be identified.

Our 3D organoid studies further support a progenitor role for *Lyz1*^+^ AT2 cells. Freshly isolated *Lyz1*^+^ AT2 cells efficiently generated spherical organoids composed of AT1 and AT2 cells when co-cultured with lung fibroblasts. Notably, *Lyz1*^+^ AT2-derived organoids were larger and contained more Ki67^+^ cells than those derived from general unselected AT2 cells, indicating superior proliferative capacity. Consistently, pathway analyses of scRNA-seq data exhibited enriched rRNA processing, ribosome assembly and translation in *Lyz1*^+^ AT2 cells. These properties seem to parallel those of CD44^high^ AT2 cells, which form larger organoids and more efficiently generate AT1 cells than CD44^low^ AT2 cells [18]. Our scRNA-seq analysis showed that a few *Lyz1*^+^ AT2 cells express CD44 at high levels, but it remains unclear whether these cells overlap functionally with the CD44^high^ progenitor population. Likewise, PD-L1^high^ SPC^low^ AT2 cells were identified as progenitor cells with a significant expansion capability in a pneumonectomy lung injury model [16]. This subpopulation exhibits a higher organoid formation efficiency and generates larger organoids than PD-L1^low^ SPC^high^ mature AT2 cells [16]. Notably, our scRNA-seq analysis revealed minimal PD-L1/CD274 expression in AT2 or other lung epithelial cells. Thus, *Lyz1*^+^ AT2 cells likely represent a unique progenitor subset, distinct from both CD44^high^ and PD-L1^high^ SPC^low^ AT2 subpopulations.

In summary, we identified an AT2 progenitor cell subpopulation defined by exclusive *Lyz1* expression. Genetic lineage tracing demonstrated that this unique subpopulation contributes to lung regeneration in two lung injury models. They also exhibit a higher proliferative potential compared to general AT2 cells. Our findings therefore, expand the understanding of AT2 cell heterogeneity and highlight *Lyz1*^+^ AT2 cells as a distinct progenitor population critical for alveolar repair.

## 4. Materials and Methods

### 4.1. Mice

The *Lyz1*^*3′UTR-IRES-CreERT2*^ (*Lyz1*^*CreERT2*^) [41] and *Sftpc*^*CreERT2*^ [42] mouse strains have been previously described. *Rosa26*^*tdTomato*^ (Jax# 007914) reporter mice were obtained from The Jackson Laboratory. All mice used in this study were maintained on a C57BL/6 background, and both males and females aged 8–12 weeks were included in experiments. Animals were housed in the animal facility at Columbia University Medical Center, with a 12-h light/dark cycle, controlled temperature (18–23 °C), and humidity (40–60%). All animal experiments and husbandry procedures were performed in accordance with protocols approved by the Institutional Animal Care and Use Committee (IACUC) at Columbia University.

### 4.2. Tamoxifen Administration

To induce Cre recombinase activity in *Lyz1*^*CreERT2*^ and *Sftpc*^*CreERT2*^ mice, tamoxifen (Sigma, St. Louis, MI, USA, T5648) was dissolved in corn oil and administered by intraperitoneal injection at 200 mg/kg body weight at the indicated time points.

### 4.3. Lung Injury Mouse Models

Influenza virus-induced lung injury was established as previously described [43]. Briefly, the influenza A/Puerto Rico/8/1934 H1N1 (PR8) virus was diluted in DMEM, and 250 plaque-forming units (pfu) were administered intranasally to anesthetized mice. Bleomycin-induced lung injury was performed as described previously [44]. In brief, mice were anesthetized and given an intratracheal injection of bleomycin sulfate (Fresenius Kabi, Lake Zurich, IL, USA, USP) dissolved in sterile saline at 1.75 U/kg body weight using a 30-gauge needle. Lung tissues were collected at the indicated time points for downstream analyses.

### 4.4. Tissue Preparation and Histology

Mice were euthanized by isoflurane overdose, and lungs were inflated with 4% paraformaldehyde (PFA) and fixed at 4 °C overnight. For paraffin embedding, fixed lungs were washed with PBS and then dehydrated through a graded ethanol series, cleared in Histoclear solution, and embedded in paraffin wax. Serial sections (7 µm) were cut and collected for histological and immunofluorescence analyses. Hematoxylin and eosin (H&E) staining was performed following standard protocols previously described [44]. Histological images were acquired using a Leica DM4 B microscope.

### 4.5. Immunofluorescence Staining

Paraffin-embedded sections were processed for immunostaining as described previously [22,44]. In brief, sections were dewaxed, rehydrated through a graded ethanol series, and subjected to antigen retrieval by high-pressure heating in antigen unmasking solution (Vector Laboratories, Newark, CA, USA, H-3300) for 2 min. After washing with PBS, sections were permeabilized and blocked for 1 h at room temperature in blocking buffer (0.2% Triton X-100 and 5% normal donkey serum in PBS. Primary antibodies were diluted in blocking buffer and applied overnight at 4 °C: anti-Lysozyme (BioGenex, Fremont, CA, USA, AR024–10R, ready to use), anti-tdtomato (biorbyt, Cambridge, UK, orb182397, 1:1000), anti-ProSPC (Abcam, Cambridge, UK, ab211326, 1:500), anti-Krt8 (DSHB, Iowa city, IA, USA, TROMA-I, 1:200), anti-Pdpn (DSHB, 8.1.1, 1:500), anti-SCGB1A1 (Santa Cruz Biotechnology, Dallas, TX, USA, sc-365992, 1:50), anti-Ki67 (Cell Signaling Technology, Danvers, MA, USA, 9129S, 1:200), anti-Krt5 (BioLegend, San Diego, CA, USA, 905901, 1:500) and anti-Hopx (Santa Cruz, sc-398703, 1:50). Following three washes with PBS, sections were incubated with fluorophore-conjugated secondary antibodies for 2 h at room temperature, counterstained with DAPI, and mounted in Fluoromount-G (SouthernBiotech, Birmingham, AL, USA, 0100-20). Images were acquired using a Zeiss LSM T-PMT confocal laser scanning microscope.

### 4.6. Isolation of Mouse AT2 Cells

Mouse AT2 cells were isolated as previously described with modifications [45]. Briefly, lungs were inflated with Dispase II digestion buffer (15 mg/mL) and incubated at room temperature for 45 min. Tissues were minced with a sterile razor blade and digested in DMEM containing DNase I (10 U/mL) for 10 min at 37 °C. The resulting suspension was sequentially filtered through 100-µm and 40-µm strainers. Cells were pelleted by centrifugation at 300 g for 5 min, resuspended in red blood cell lysis buffer (Sigma), incubated for 2 min at 37 °C, washed in HBSS containing 10% FBS, and centrifuged again at 300 g for 5 min. For flow cytometric isolation of tdTomato^+^ AT2 cells, single-cell suspensions were incubated with PE-Cy7-CD45 (Biolegend, 103114) and APC-EpCAM (Biolegend, 118214) antibodies in FACS buffer (5% FBS, 0.5mM EDTA in PBS) for 1 h at 4 °C. Cells were then incubated with a Live/Dead dye for 10 min at room temperature to exclude dead cells. After washing in FACS buffer, live CD45⁻ EpCAM^+^ tdTomato^+^ AT2 cells were sorted using a BD Influx cell sorter.

### 4.7. Mouse AT2-Fibroblast 3D Organoid Culture

3D organoid co-culture of AT2 cells and fibroblasts was performed as previously described with modifications [46]. Briefly, 3000 freshly isolated tdTomato^+^ AT2 cells from *Lyz1*^*CreERT2*^;*R26*^*tdT*^ or *Sftpc*^*CreERT2*^;*R26*^*tdT*^ mice were mixed with 2 × 10^5^ Mlg2908 mouse lung fibroblasts (ATCC) in 100 μL of growth factor-reduced Matrigel (Corning, Corning, NY, USA). The mixture was seeded into 24-well Transwell inserts and incubated at 37 °C with 5% CO_2_ for 30 min to allow Matrigel polymerization. After solidification, 410 μL of culture medium (DMEM/F12 supplemented with 10% Active FBS, 1× Insulin-Transferrin-Selenium, 10 μM SB431542, 100 U/mL Penicillin-Streptomycin) was added to the lower chamber. Medium was replaced every other day. Organoids were imaged using a Leica DMi8 inverted microscope with z-Stack. At the indicated time points, organoids were fixed with 4% PFA and subsequently processed for histological analysis and immunostaining.

### 4.8. Single Cell RNA Sequencing (scRNA-Seq) Analysis

Publicly available scRNA-seq datasets of normal mouse lung epithelial cells (GSE171571, GSE132910, GSE138585 and GSE202226) were downloaded from NCBI Gene Expression Omnibus (GEO). Raw count matrices were imported into Seurat (v4.4.0) for analysis. Low-quality cells were excluded if they expressed fewer than 200 genes, more than 6000 genes, or if >20% of total transcripts were mitochondrial in origin. Data normalization and variable feature selection were performed using the NormalizeData and FindVariableFeatures functions. To integrate datasets and correct for batch effects, Seurat objects were merged and processed with the RunFastMNN function. Dimensionality reduction was performed using ScaleData, principal component analysis (PCA), and Uniform Manifold Approximation and Projection (UMAP). Cell clustering was carried out with the FindNeighbors and FindClusters functions. Differentially expressed genes for each cluster were identified with FindAllMarkers under default parameters. Clusters were manually annotated according to established marker gene expression. For AT2 sub-clustering, *Sftpc*^+^ clusters were selected and re-analyzed, revealing three distinct populations: canonical AT2 cells, *Lyz1*^+^ AT2 cells, and proliferating AT2 cells, defined by their respective marker gene signatures. Differentially expressed genes (DEGs) between AT2 cells and *Lyz1*^+^ AT2 cells identified with FindMarkers under default parameters were used for Gene Ontology (GO) analysis and Gene Set Enrichment Analysis (GSEA).

### 4.9. Quantification and Statistical Analysis

For quantification of tdTomato^+^ cells and SPC^+^ cells, at least 10 randomly selected fields (20× magnification) were captured, and positive cells were manually counted using ImageJ software (version 1.51). Organoid diameter and number were measured with Leica LAS X software (version 3.7.4.23463). Each experimental group included at least three biological replicates. Data are presented as means ± s.e.m., and statistical analyses were performed using GraphPad Prism 8. Comparisons between two groups were assessed using an unpaired two-tailed *t*-test with Welch’s correction. A *p* value < 0.05 was considered statistically significant.

## Supplementary Material

Supplementary Information The following supporting information can be found at: https://www.sciepublish.com/article/pii/774, Figure S1: scRNA-seq reveals distinct lung epithelial populations. (**A**) UMAP plot showing distinct epithelial cell populations in adult mouse lungs. Re-analysis of the publicly available datasets GSE171571, GSE132910, GSE138585 and GSE202226. (**B**) Dot plot showing the representative markers for each lung epithelial cell population. (**C**–**E**) UMAP plot showing the expression of *Axin2* (**C**), *Cd44* (**D**) and *Cd274* (**E**) in adult mouse AT2 cell populations. (**F**,**G**) The top 10 GO terms (**F**) and GSEA pathways (**G**) associated with genes enriched in *Lyz1*^*+*^ AT2 subpopulation; Figure S2: *Lyz1*-expressing AT2 cells contribute to lung regeneration upon bleomycin treatment. (**A**) Schematic of tamoxifen injection and bleomycin challenge of *Lyz1*^*CreERT2*^;*R26*^*tdT*^ mice. (**B**) Representative images showing SPC^+^tdTomato^+^ cells in lung tissues. (**C**) Representative images showing Krt8^+^tdTomato^+^ cells in bleomycin-challenged lung tissues. Magnified image of dashed square frame showing on the right. (**D**) Representative images showing Pdpn^+^tdTomato^+^ cells (arrows) in bleomycin-challenged lung tissues. Data are representative of at least three independent experiments. Scale bars: 100 μm. Table S1: Mouse_AT2_Cell_subpopulation_Markers.

## Data Availability

The scRNA-seq datasets (GSE171571, GSE132910, GSE138585 and GSE202226) were publicly available in NCBI Gene Expression Omnibus (GEO).
