# Supplementary material for: Lyz1-Expressing Alveolar Type II Cells Contribute to Lung Regeneration
Source: J Respir Biol Transl Med. Author manuscript; Available in PMC 2026 Jan 16. (PMC12807509; doi:10.70322/jrbtm.2025.10011)
Supplement: Supplementary Information — The following supporting information can be found at: https://www.sciepublish.com/article/pii/774, Figure S1: scRNA-seq reveals distinct lung epithelial populations. (A) UMAP plot showing distinct epithelial cell populations in adult mouse lungs. Re-analysis of the publicly available datasets GSE171571, GSE132910, GSE138585 and GSE202226. (B) Dot plot showing the representative markers for each lung epithelial cell population. (C–E) UMAP plot showing the expression of Axin2 (C), Cd44 (D) and Cd274 (E) in adult mouse AT2 cell populations. (F,G) The top 10 GO terms (F) and GSEA pathways (G) associated with genes enriched in Lyz1+ AT2 subpopulation; Figure S2: Lyz1-expressing AT2 cells contribute to lung regeneration upon bleomycin treatment. (A) Schematic of tamoxifen injection and bleomycin challenge of Lyz1CreERT2;R26tdT mice. (B) Representative images showing SPC+tdTomato+ cells in lung tissues. (C) Representative images showing Krt8+tdTomato+ cells in bleomycin-challenged lung tissues. Magnified image of dashed square frame showing on the right. (D) Representative images showing Pdpn+tdTomato+ cells (arrows) in bleomycin-challenged lung tissues. Data are representative of at least three independent experiments. Scale bars: 100 μm. Table S1: Mouse_AT2_Cell_subpopulation_Markers. [file NIHMS2126477-supplement-Supplementary_Information.zip › Figures S1 and S2.docx]

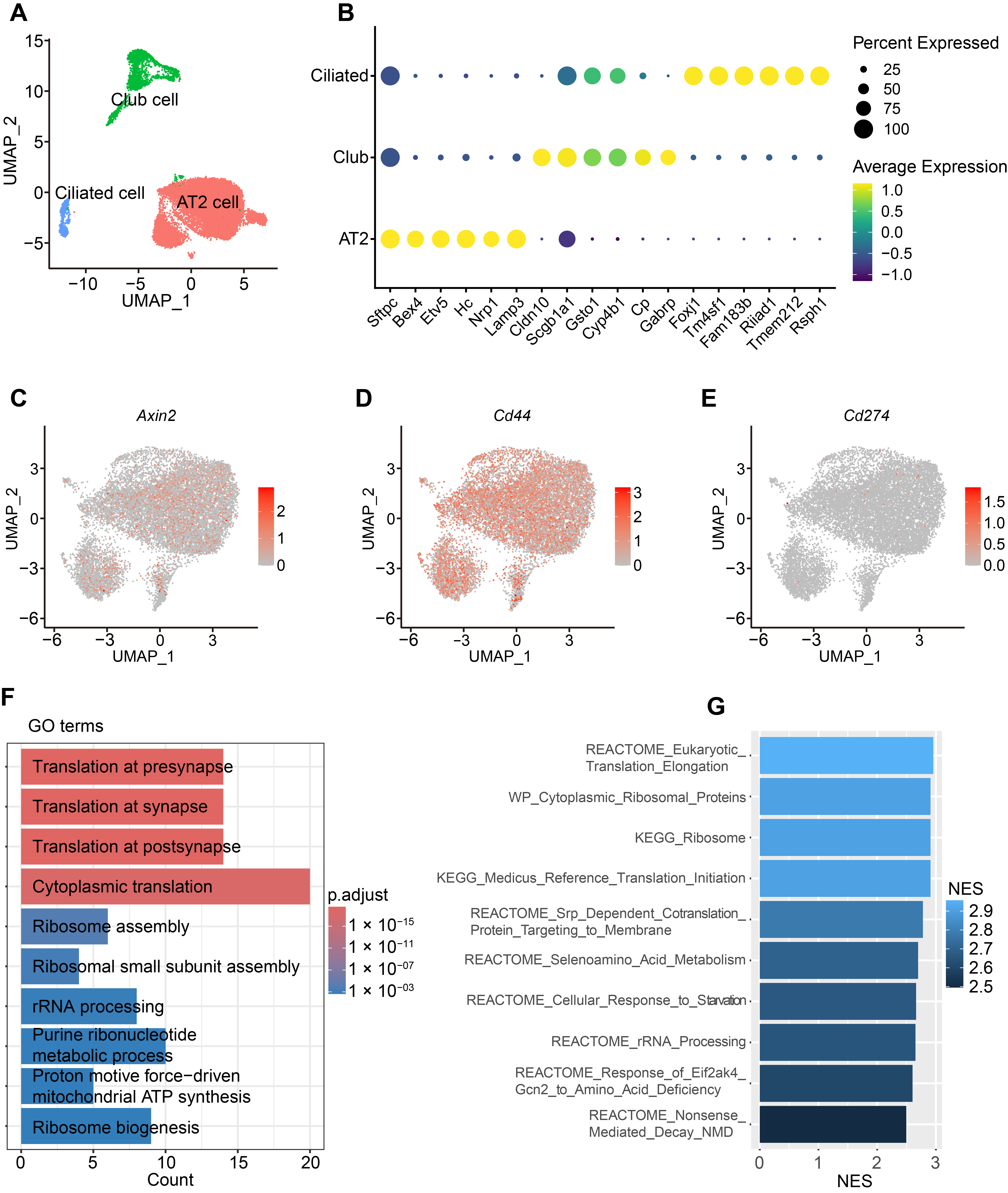


**Figure S1.** scRNA-seq reveals distinct lung epithelial populations. (**A**) UMAP plot showing distinct epithelial cell populations in adult mouse lungs. Re-analysis of the publicly available datasets GSE171571, GSE132910, GSE138585 and GSE202226. (**B**) Dot plot showing the representative markers for each lung epithelial cell population. (**C**,**E**) UMAP plot showing the expression of *Axin2* (**C**), *Cd44* (**D**) and *Cd274* (**E**) in adult mouse AT2 cell populations. (**F**,**G**) The top 10 GO terms (**F**) and GSEA pathways (**G**) associated with genes enriched in *Lyz1*^+^ AT2 subpopulation.


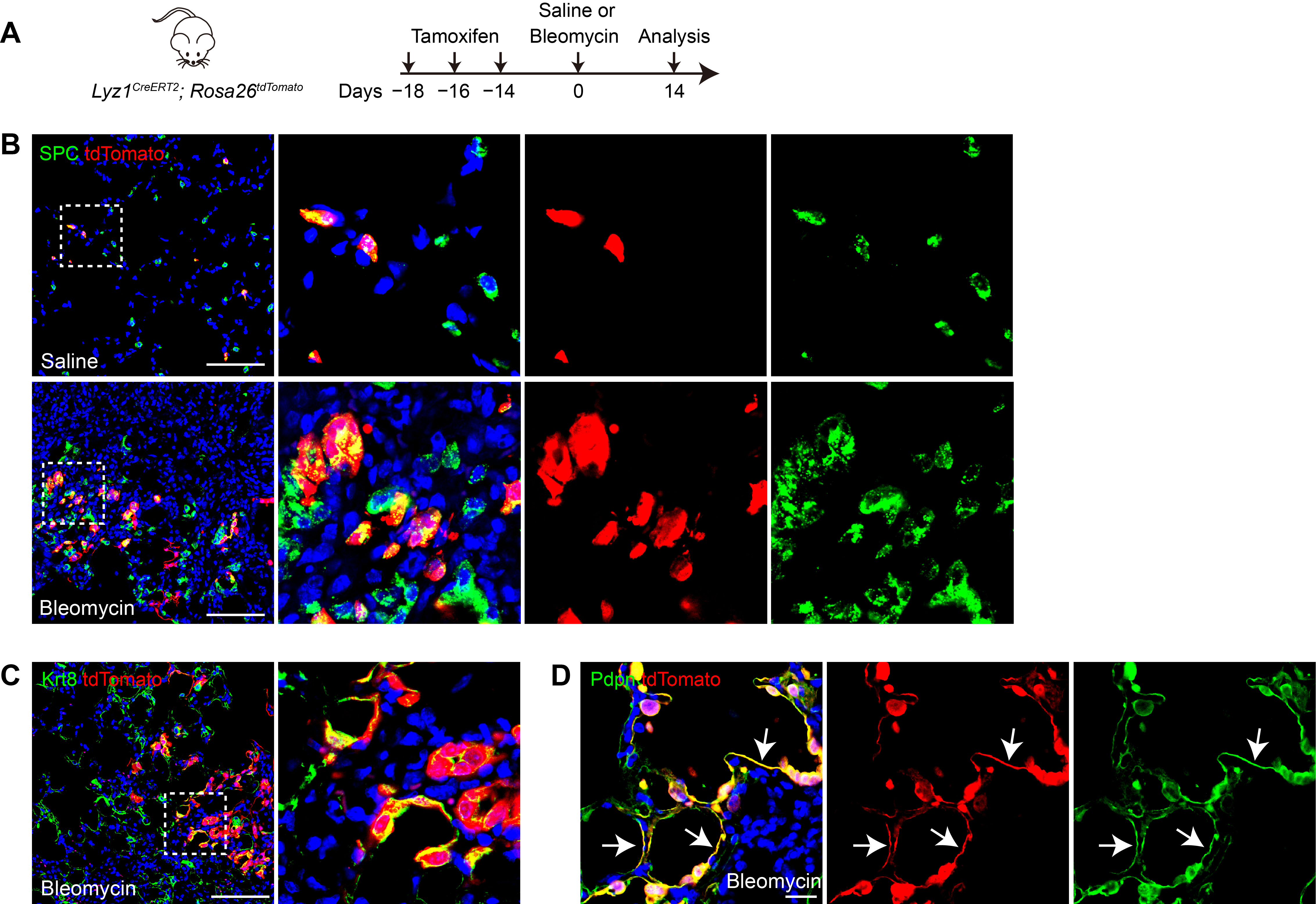


**Figure S2.** *Lyz1*-expressing AT2 cells contribute to lung regeneration upon bleomycin treatment. (**A**) Schematic of tamoxifen injection and bleomycin challenge of *Lyz1^CreERT2^*;*R26^tdT^* mice. (**B**) Representative images showing SPC^+^tdTomato^+^ cells in lung tissues. (**C**) Representative images showing Krt8^+^tdTomato^+^ cells in bleomycin-challenged lung tissues. Magnified image of dashed square frame showing on the right. (**D**) Representative images showing Pdpn^+^tdTomato^+^ cells (arrows) in bleomycin-challenged lung tissues. Data are representative of at least three independent experiments. Scale bars: 100 μm.
